# Prevalence of chronic kidney disease associated with cardiac and vascular complications in hypertensive patients: a multicenter, nation-wide study in Thailand

**DOI:** 10.1186/s12882-017-0528-3

**Published:** 2017-04-03

**Authors:** Rungroj Krittayaphong, Ram Rangsin, Bandit Thinkhamrop, Cameron Hurst, Suthee Rattanamongkolgul, Nintita Sripaiboonkij, Wipaporn Wangworatrakul

**Affiliations:** 1 Division of Cardiology, Department of Medicine, Siriraj Hospital, Mahidol University, 2 Wanglang Road, Bangkoknoi, Bangkok, 10700 Thailand; 2 Department of Military and Community Medicine, Phramongkutklao College of Medicine, Bangkok, Thailand; 3 Faculty of Public Health, Khon Kaen University, Khon Kaen, Thailand; 4 Faculty of Medicine, Chulalongkorn University, Bangkok, Thailand; 5 Department of Preventive and Social Medicine, Srinakarinwirot University, Nakornnayok, Thailand; 6 Cancer Registry Unit, Ramathibodi Hospital, Mahidol University, Bangkok, Thailand

**Keywords:** Chronic kidney disease, Hypertension, Cardiac, Vascular, Complication

## Abstract

**Background:**

Hypertension and chronic kidney disease (CKD) are common conditions and both are major risk factors for cardiovascular events. The objectives were 1) to study the prevalence of CKD in hypertensive patients and 2) to study the association of CKD with cardiac and vascular complications in a multicenter, nation-wide fashion.

**Methods:**

This cross-sectional study evaluated patients aged 20 years or older who were diagnosed with hypertension and who had been treated for at least 12 months at 831 public hospitals in Thailand during the 2012 study period. Outcome measurements included calculated glomerular filtration rate (GFR) and cardiac and vascular complications that included coronary artery disease, stroke, peripheral arterial disease, heart failure, and atrial fibrillation. Multivariable modeling was conducted to determine independent factors associated with increased risk of cardiac and vascular complications.

**Results:**

A total of 28770 patients were enrolled. Average age was 62.8 years and 37% were male. Prevalence of CKD stage 3 and 4–5 was 33.2 and 4.3%, respectively. Prevalence of cardiac and vascular complications was 10.5% (5% having coronary artery disease, 3.9% stroke, 1.7% heart failure, and 1.2% atrial fibrillation). CKD was an independent risk factor associated with each of the complications and overall cardiac and vascular complications with an adjusted Odds ratio of 1.4 for CKD stage 3 and 1.9 for CKD stage 4–5.

**Conclusion:**

Prevalence of CKD stage 3–5 in hypertensive population was 37.5%. CKD is an independent risk factor for adverse cardiac and vascular outcome.

## Background

Chronic kidney disease (CKD) is a leading cause of morbidity and mortality among patients around the world [1]. Prevalence of CKD has been estimated to be 8–16% worldwide [1]. Diabetes and hypertension are major risk factors for the development of CKD [1, 2]. Hypertension doubles the risk of CKD in the general population and the risk ratio has been reported to be as high as 3.9 in older population [3]. Control of hypertension can reduce the risk of developing CKD [2].

CKD is classified into 5 stages; CKD stages 1 and 2 have a glomerular filtration rate (GFR) of higher than 60 ml/min. The GFRs of CKD stages 3, 4 and 5 are 30–60, 15–30 and < 15 ml/min respectively [4]. Prevalence of the different stages of CKD in Thailand was reported in 2 major studies namely the National Health Exam Survey (NHES) [5] and the Thai Screening and Early Evaluation of Kidney Disease (Thai SEEK) study [6]. The NHES study involved 3117 subjects with the average age of 33.6 years; 22.5% of subjects had hypertension [5]. Prevalence of CKD stages 3, 4, and 5 from the NHES studies was 8.1, 0.2, and 0.15%, respectively. The Thai SEEK study enrolled 3459 subjects with mean age 46.2 years, 16.5% of patients had a history of hypertension. The Thai SEEK study found prevalence of CKD stages 1–2 and stage 3–4 of 8.9 and 8.6%, respectively [6]. However, there are limited data on the prevalence of CKD in hypertensive population is Asian countries.

Recent practice guidelines for cardiovascular prevention regarded CKD as a major risk factor for cardiovascular event [7]. CKD stages 4–5 increases risk of cardiovascular event with an Odds ratio of 4–50 [8]. Even in patients with cardiac disease, severity of CKD has been shown to be a better predictor of cardiovascular events than left ventricular ejection fraction [9, 10]. Prevalence of CKD among patients admitted with acute myocardial infarction was 30–42% [11]. Patient with CKD had an increased risk of cardiovascular event 2–7 times higher than those without CKD [8, 11]. There was little data on the association of CKD and cardiovascular outcome in Asian population [12]. It has been reported that patients with CKD are normally more likely to receive treatment that does not closely follow generally accepted practice guidelines than treatment received by patients that do not have CKD [11].

The primary objective of this study was to determine the association between CKD and cardiac and vascular complications in hypertensive patients. The secondary objectives were to: 1) study the prevalence of CKD in hypertensive patients; and 2) study the prevalence of cardiac and vascular complications in patients with hypertension.

## Methods

### Study population

This cross-sectional survey was conducted in Thailand in 2012 to evaluate nation-wide standard of care and treatment outcome among hypertensive patients who were treated at public Thailand Ministry of Public Health (MoPH) hospitals in Thailand and private clinics in Bangkok that participate in the Thailand National Health Security Office (NHSO)’s program. Patients were enrolled if they were diagnosed with hypertension, aged 20 years or older and received regular treatment in the participating hospital or clinic for the past 12 months. All patients were recruited from the ambulatory unit. We excluded patients participating in clinical trials.

We used a two-stage stratified cluster, proportional to the size sampling technique, to select a nationally and provincially representative sample of patients who were diagnosed as hypertension. In provinces outside of Bangkok, participating hospitals included all public hospitals under the oversight of the Thailand Ministry of Public Health (MoPH). For Bangkok, targeted hospitals included all hospitals and clinics that were participated in the Thailand NHSO program. The sampling started at the first stage of 77 strata at the provincial level. The second stage was at the hospital level in each province. In each province, hospitals were stratified into 5 strata, according to sizes. The largest size was more than 500 beds (regional center hospital) followed by 200–500 beds (provincial general hospital), 90–120 beds (large community hospital), 30–90 beds (medium community hospital), and 10–30 beds (small community hospital). University hospitals were not included from our study.

This study was approved by the Royal Thai Army Medical Department Ethical Review Board, the Ethical Review Committee for Research in Human Subjects, Thailand Ministry of Public Health. The protocol for this study was also approved by the Institutional Review Boards of all participating hospitals. Written informed consent was obtained from subjects prior to participation. This study was supported by the Thai National Health Security Office (NHSO).

### Data collection process

A total of 831 hospitals under the Thai Universal Coverage Scheme were included. The number of regional hospitals, general hospitals and community hospitals were 25, 70, and 736 respectively. Every regional and general hospital was included. Community hospitals were randomly sampled with 70% of small-sized hospitals, 20% of medium-sized hospitals and 10% of large-size community hospitals. These proportions were based on the proportion of health care provided at various hospitals levels. Patients diagnosed as hypertension were randomly selected according to the proportion that were registered at each hospital. Sample size of the study was derived from the proportion to size model.

To minimize the selection bias, all patients who were randomly selected was invited by the study nurse to participate in this study. Data were retrieved from patient’ medical records, which included baseline characteristics, presence of hypertensive complications, and laboratory test results. The study nurse was trained for the process of data collection. The required data were retrieved from medical recorded and entered in the case record form (CRF). Entry of data in the CRF was based on data that were written in the medical record or from the ICD-10 diagnostic code. This applied to diagnosis of hypertension and all other diagnoses. A majority of participating hospitals had medical record in non-electronic format limiting the opportunity of data transfer from hospital electronic database. The case record form was then sent to the central data management or MEDRESNET (Medical Research Network). Data management officers at MEDRESNET manually adjudicated the data and ensured that the data in the case record form complied with the study protocol. In case of doubt the query was sent to study site to verify with the data in the medical record.

### Assessment of renal function

Glomerular filtration rate (GFR) was calculated by Chronic Kidney Disease Epidemiology Collaboration (CKD-EPI) formula [13, 14]. GFR was classified into 3 groups: ≥60 ml/min; 30–59 ml/min (CKD stage 3); and, <30 ml/min (CKDs stage 4 or 5).

### Other measurements

The following variables were collected: demographic data; height; weight; body mass index (BMI); cardiovascular risk factors; systolic blood pressure (SBP); diastolic blood pressure (DBP); blood chemistry, including fasting plasma glucose (FPG), lipid profiles, serum creatinine, uric acid, proteinuria from urine analysis; available ECG data and results. The laboratory results were the most recent results within 12 months prior to the consent process.

The data management team is responsible for inquiries to study sites to verify data. By a random selection process, site monitoring was performed in approximately 60 hospitals or 10% of study sites.

Primary outcome measurements were cardiac and vascular complications, which included coronary artery disease (CAD), stroke, peripheral arterial disease (PAD), heart failure, and atrial fibrillation. CAD was defined as angina pectoris, myocardial infarction, or coronary revascularization. Stroke was defined as cerebral infarction, TIA, unspecified stroke, or hemorrhagic stroke. Vascular complication was defined as CAD, stroke, or PAD. Secondary outcomes were defined as individual components of cardiac and vascular complications.

### Statistical analysis

Continuous data were presented as mean and standard deviation and categorical variables were expressed as number and percentage. Comparisons of continuous data were performed by independent samples *t*-test. Comparisons were performed by chi-square test for categorical data. Continuous data were grouped for Odds ratio analysis, as follows: age ≥ or < 65 years, SBP ≥ or < 140 mmHg, DBP ≥ or < 90 mmHg, TC ≥ or < 200 mg/dl, TG ≥ or < 200 mg/dl, HDL < or ≥ 40 mg/dl, LDL ≥ or < 100 mg/dl, BMI ≥ or < 25 kg/m2, GFR < or ≥ 60 ml/min, and uric acid ≥ or < 7 mg/dl in male and ≥ or < 6 mg/dl in female. When missing data were detected during data entry, query of raw data was sent to the study site for verification. During data analysis, since the number of patients is large and number of missing data was small in the majority of data as shown in Table 2 (meaning that bivariate analysis represents an available case analysis). For the multivariable models complete-cases analysis and multiple imputation analysis were performed. We have now included a forest plot in the results that indicates the effect on missing data. This plot allows comparison between the estimates and 95% CI from the complete cases and imputed analysis.

Odds ratio and 95% confidence interval (CI) for univariate analysis were determined by chi-square test. Multivariate logistic regression analysis (with forward LR) was used to determine the independent factors that are associated with increased risk of cardiac and vascular complications using complete-cases analysis with and without imputation method for the missing data. For imputation method, missing values were imputed by using the means of complete cases with noise added based on the t-distribution. CKD was tested to determine whether or not it was an independent risk factor associated with cardiac and vascular complications after adjustment for other baseline characteristics. A *p*-value less than 0.05 was considered statistically significant. All statistical analyses were performed using SPSS Statistics version 20 (SPSS, Inc., Chicago, IL, USA).

## Results

**Fig. 1:**
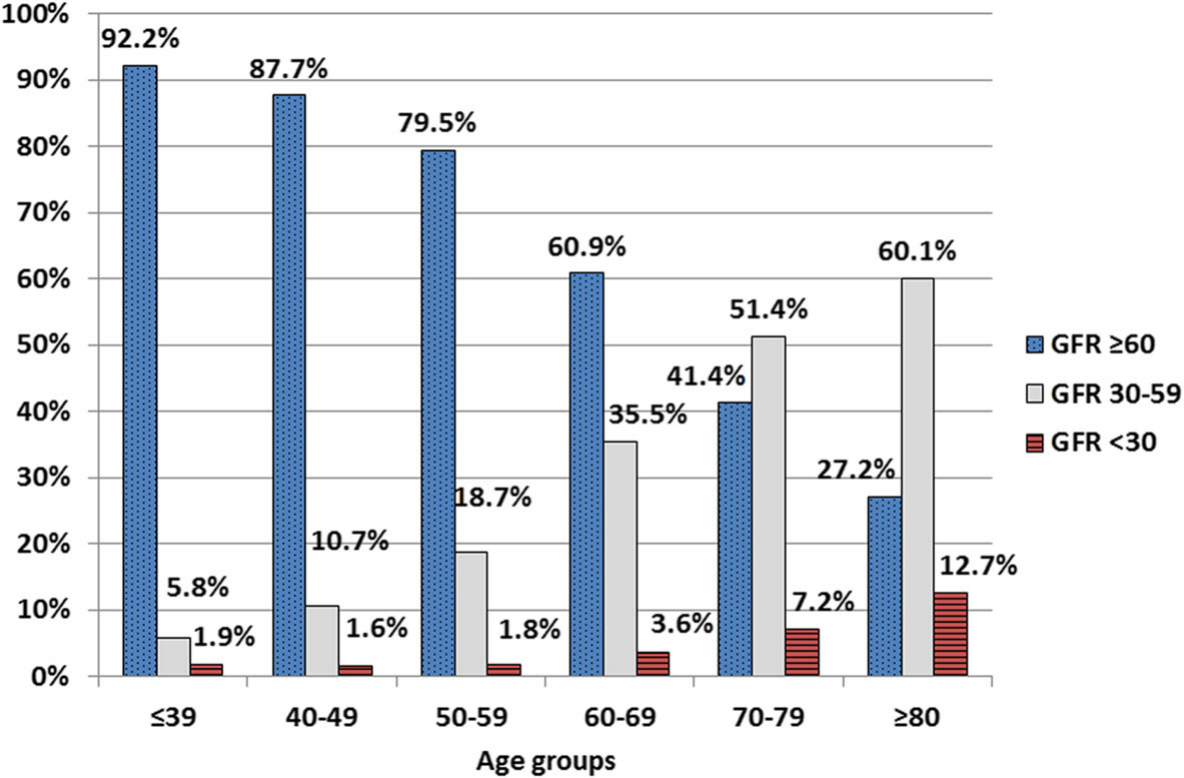
Prevalence of CKD by age groups

**Fig. 2:**
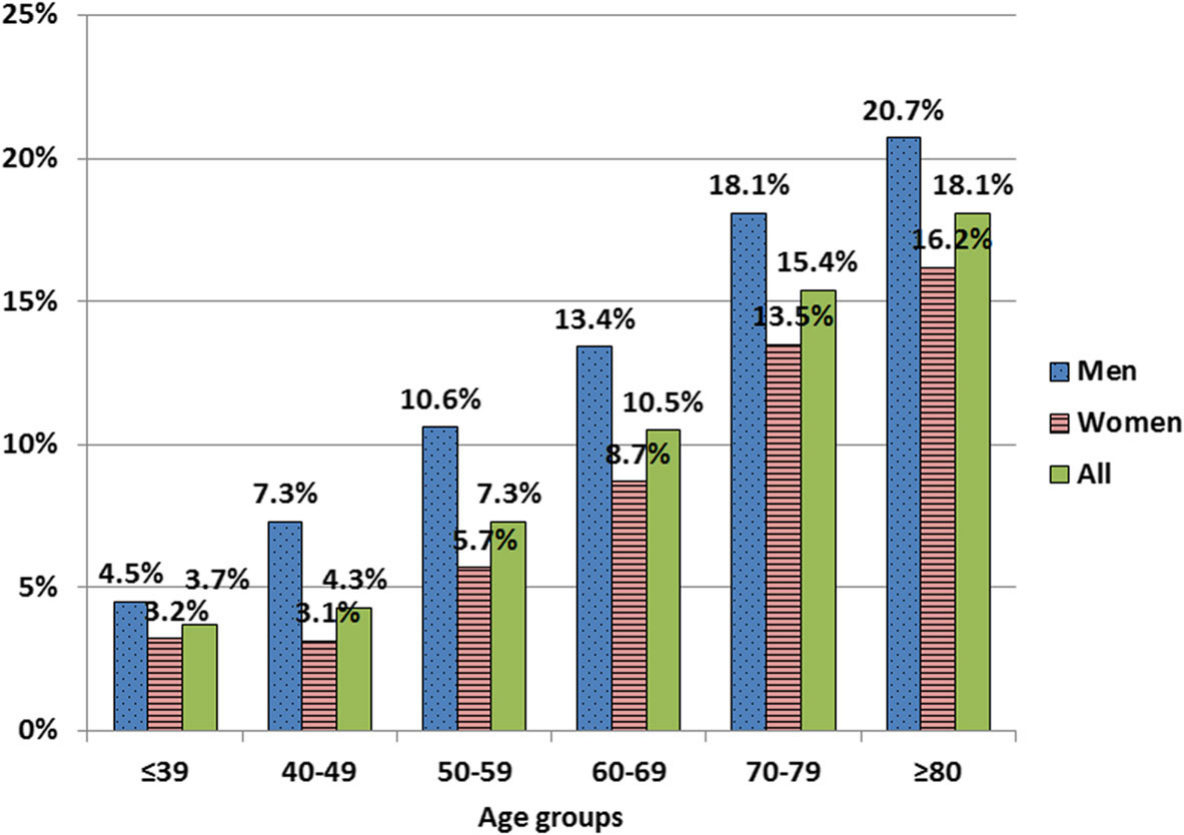
Prevalence of vascular and cardiac complications in men and women by age groups

**Fig. 3:**
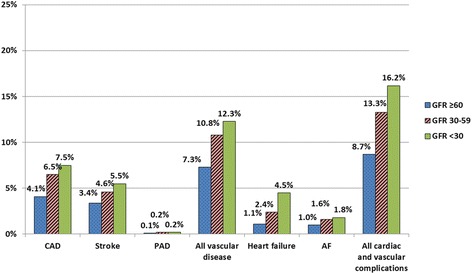
Prevalence of each type of vascular and cardiac complication by GFR groups

There were a total of 28770 patients; with a mean age 62.8 ± 11.3 years; 10650 (37.0%) of our subjects were male. Average GFR was 68.3 ± 22.8 ml/min; ≥60 ml/min in 17970 (62.5%), 30–59 ml/min in 9553 (33.2%), and <30 ml/min in 1247 patients (4.3%). Prevalence of CKD stage 3 and stages 4–5 increased with age (Fig. 1) but appeared to have no gender-related predisposition (CKD stage 3 and stages 4–5 were 35.3 and 4.3% in men and 32.0% and 4.4% in women). Prevalence of cardiac and vascular complications is shown in Table 1. Overall prevalence was 10.5% with CAD being the most prevalent condition followed by stroke. Cardiac and vascular complications increased with age and men more than women (Fig. 2). Prevalence of all cardiac and vascular complications appears to increase as the level of GFR declined (Fig. 3).

**Table 1:** Prevalence of cardiac and vascular complications

Cardiac and vascular complications	Number (%)
	(Total *N* = 28770)
Coronary artery disease	1442 (5.0)
Stroke	1118 (3.9)
Peripheral arterial disease	29 (0.1)
All vascular disease	2499 (8.7)
Heart failure	487 (1.7)
Atrial fibrillation	346 (1.2)
All cardiac and vascular complications	3031 (10.5)

**Fig. 4:**
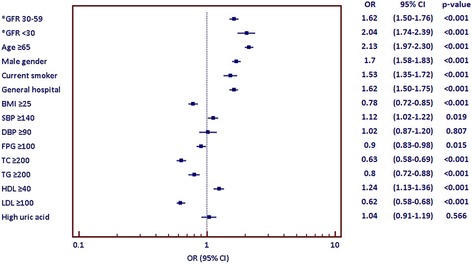
Bivariate analysis factors affecting cardiac and vascular complications (shown as Odds ratio, 95% CI, and forest plot) (*GFR was analyzed by using group with GFR ≥ 60 ml/min as the reference)

**Fig. 5:**
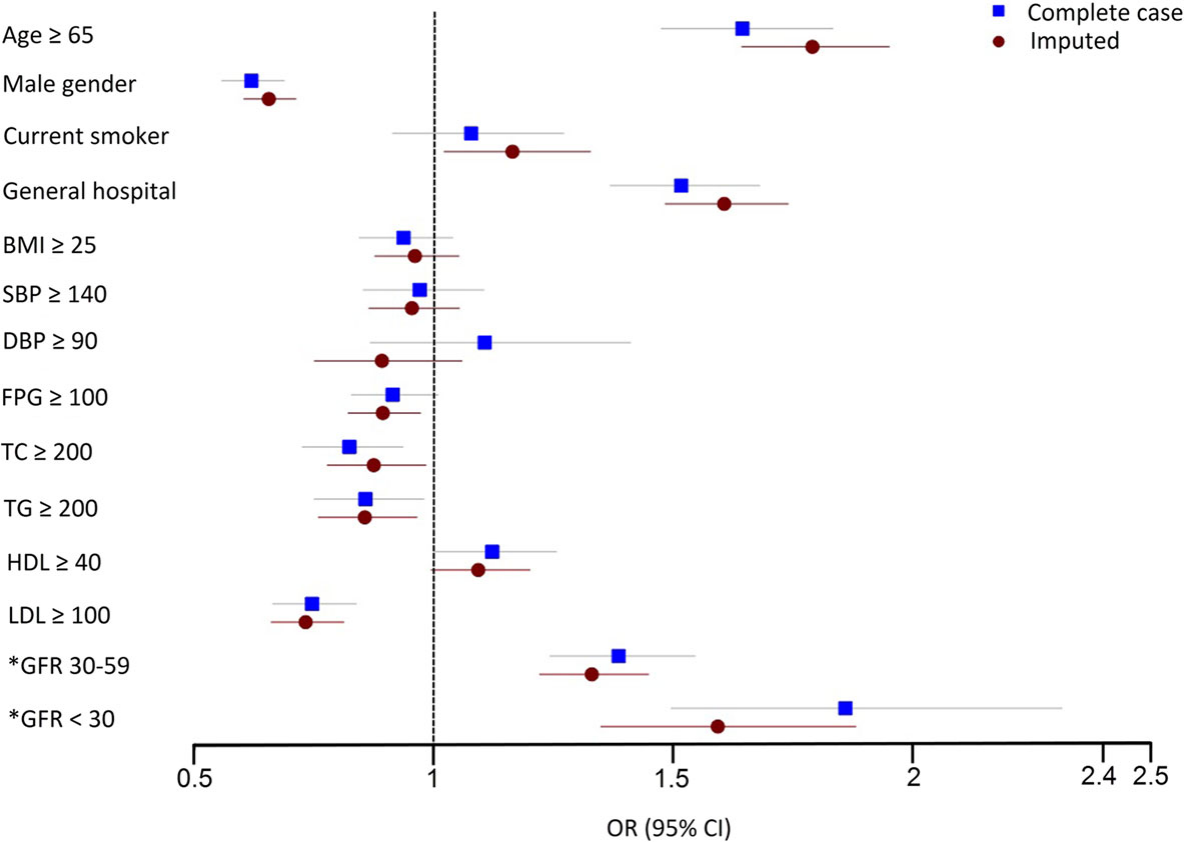
Forest plot shows Odds ratio and 95% CI of multivariate analysis of factors affecting cardiac and vascular complications from complete case analysis and imputation method (*GFR was analyzed by using group with GFR ≥ 60 ml/min as the reference)

Baseline characteristics, blood chemistry data, control status of cardiovascular risk factors and prevalence of cardiac and vascular complications in each group are shown in Table 2. Hypertension was well controlled (SBP <140 and DBP <90 mmHg) in 78.0%. FPG was in good control (<100 mg/dL) in 47.8% and control for LDL-cholesterol was good (<100 mg/dl) in 34.9%. The univariate analysis indicates that increased age, male gender, current smoking, general hospital, low BMI, high SBP, low FPG, low total cholesterol, low triglyceride, high HDL-cholesterol, low LDL-cholesterol, and low levels of GFR are associated with cardiac and vascular complications (all *p* < 0.05). Odds ratios and a forest plot indicating the association of each of the baseline characteristics with cardiac and vascular complications are shown in Fig. 4. When the selected factors from univariate analysis are mutually adjusted in the multivariable modelling, higher age, male gender, general hospital, low total cholesterol, low triglyceride, high HDL-cholesterol, low LDL-cholesterol, and low levels of GFR are identified as independently associated with cardiac and vascular complications (all *p* < 0.05). Crude and adjusted Odds ratio of factors that were independently associated with cardiac and vascular complications is shown in Table 3. Forest plot of complete-cases analysis and imputation method is shown in Fig. 5. The direction and significance of the individual effects remains largely unchanged.

**Table 2:** Patient baseline characteristics and prevalence of cardiac and vascular complications (total *N* = 28770)

Baseline variables	n	Mean ± SD/number (%)	Prevalence of cardiac and vascular complications (%)
Age (years)	28770	62.8 ± 11.3	
Gender	28770		
Male		10650 (37.0)	13.8
Female		18120 (63.0)	8.6
Current smoker	28770		
Yes		2322 (8.1)	14.7
No		26448 (91.9)	10.2
Type of hospital	27662		
General		9023 (32.6)	13.8
Community		18639 (67.4)	9.0
SBP (mmHg)	28758	131.1 ± 15.4	
≥ 140		5814 (20.2)	11.4
< 140		22914 (79.8)	10.3
DBP (mmHg)	28754	75.8 ± 10.5	
≥ 90		1690 (5.9)	10.7
< 90		27066 (94.1)	10.5
FPG (mg/dL)	25077	107.3 ± 31.0	
≥ 100		13088 (52.2)	10.0
< 100		11989 (47.8)	10.9
TC (mg/dL)	25228	193.8 ± 43.3	
≥ 200		10263 (40.7)	7.9
< 200		14965 (59.3)	11.9
TG (mg/dL)	26502	154.5 ± 86.8	
≥ 200		5477 (20.7)	8.6
< 200		21025 (79.3)	10.6
HDL (mg/dL)	23933	48.8 ± 13.7	
≥ 40		6060 (25.3)	11.8
< 40		17873 (74.7)	9.7
LDL (mg/dL)	25586	115.9 ± 36.4	
≥ 100		16653 (65.1)	8.6
< 100		8933 (34.9)	13.1
GFR (ml/min)	28770	68.3 ± 22.8	
≥ 60		17970 (62.5)	8.7
30–59		9553 (33.2)	13.3
< 30		1247 (4.3)	16.2
Uric acid (mg/dL)	9563	6.1 ± 1.6	
High		3890 (40.7)	10.5
Normal		5673 (59.3)	10.1

*SD* Standard deviation, *DM* Diabetes mellitus, *SBP* Systolic blood pressure, *DBP* Diastolic blood pressure, *TC* Total cholesterol, *TG* Triglyceride, *HDL* High density lipoprotein cholesterol, *LDL* Low density lipoprotein cholesterol, *GFR* Glomerular filtration rate by epidemiology collaboration formula

**Table 3:** Crude and adjusted Odds ratio of factors that were independently associated with cardiac and vascular complications

Variables	Crude OR (95% CI)	*p*-value	Multivariable adjusted OR (95% CI)	*p*-value
Age ≥65 years	2.13 (1.97–2.30)	<0.001	1.65 (1.48–1.83)	<0.001
Men	1.70 (1.58–1.83)	<0.001	1.62 (1.46–1.80)	<0.001
General hospital	1.62 (1.50–1.75)	<0.001	1.52 (1.37–1.68)	<0.001
TC ≥200 mg/dL	0.63 (0.58–0.69)	<0.001	0.82 (0.73–0.94)	0.003
TG ≥200 mg/dL	0.80 (0.72–0.88)	<0.001	0.86 (0.75–0.98)	0.023
HDL ≥40 mg/dL	1.24 (1.13–1.36)	<0.001	1.12 (1.00–1.26)	0.045
LDL ≥100 mg/dL	0.62 (0.58–0.68)	<0.001	0.75 (0.66–0.84)	<0.001
GFR ≥60 ml/min	reference	<0.001	reference	<0.001
30–59 ml/min	1.62 (1.50–1.76)		1.39 (1.24–1.55)	
< 30 ml/min	2.04 (1.74–2.39)		1.86 (1.50–2.31)	

See abbreviation lists in Table 2

Data on proteinuria was available in only 2281 patients (7.9%). Among patients who had available urine protein data, the results were negative or trace in 874 (38.3%), 1+ in 768 (33.7%), 2+ in 447 (19.6%), 3+ in 159 (7.0%), and 4+ in 33 (1.4%). Since the number of patients with available urine protein data was relatively small, we did not intend to run the primary outcome analysis for proteinuria. However, among patients with urine protein data, proteinuria had an Odds ratio for the association with cardiac and vascular complication of 1.20 (95% CI 0.92–1.58). The numerical association of proteinuria and cardiac and vascular complication was mainly for patients with CKD with the Odds ratio of 1.23 (95% CI 0.84–1.1.80) compared to those without CKD (Odds ratio 1.00, 95% CI 0.67–1.49).

**Fig. 6:**
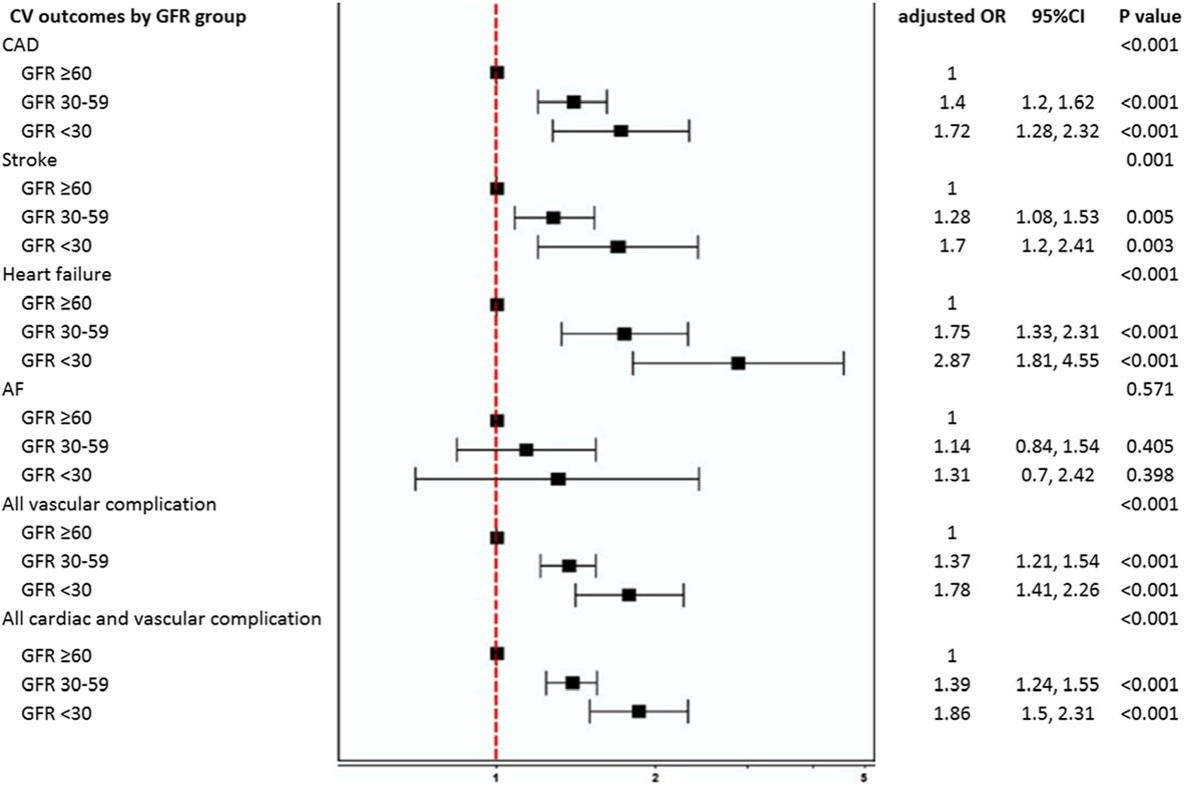
Multivariable relationships between GFR groups and each type of cardiac and vascular complication

Multivariable adjusted Odds ratio and 95% CI for association of levels of GFR with each factor of cardiac and vascular complication are shown in Fig. 6. Results of the analysis showed that the prevalence of each factor of cardiac and vascular complication increased as the level of GFR declined. Multivariable adjusted Odds ratio showed that the level of association between levels of GFR and each of cardiac and vascular complication remained the same with the exception of atrial fibrillation.

## Discussion

The results of this study demonstrated prevalence of CKD stage 3 and 4–5 in patients with hypertension to be 33.2 and 4.3%, respectively. A decline in GFR in patients with hypertension was independently associated with cardiac and vascular complications which included CAD, stroke, PAD, and heart failure.

Prevalence of CKD has increased over the past 20 years [1] as a result of greater proportion of older people in the population and a corresponding increase in the prevalence of cardiovascular risk factors, such as diabetes and hypertension [1]. Uncontrolled hypertension is a major risk factor for CKD and patients with CKD have an increased risk of hypertension not only from related stiffening of arteries, but also related to volume overload. A world-wide study showed that the prevalence of CKD significantly increases in patients at high risk for cardiovascular disease such as hypertension [15]. Prevalence of left ventricular hypertrophy was as high as 50% in patients with GFR less than 30 ml/min [16]. Treatment of hypertension is very effective in the prevention of CKD in patients with hypertension [17]. Blood pressure target in patients with CKD has been proposed to be more stringent than in patients without CKD [18].

Our study showed prevalence of CKD stage 3 and stages 4–5 in patients with hypertension of 33.2 and 4.3% respectively. Mean age in our study population was 62.8 years. Previous study in healthy Thai individuals from the NHES study showed prevalence of CKD stage 3 and stages 4–5 of 8.1 and 0.35%, respectively [5]. Data from the Thai SEEK study reported prevalence of CKD stages 3 and 4 of 7.5 and 1.1%, respectively [6]. The difference between our study and other studies mentioned here is mainly the health status of the study population. We studied patients with hypertension while previous studies focused on healthy populations. Prevalence of CKD stage 3–5 in patients with hypertension was reported to be approximately 23% [19]. However, our estimate of the prevalence of CKD in the hypertensive population was considerably higher. Although previous studies and our study enrolled patients from primary care centers, the hospitals involved in our study included larger ‘regional hospitals’. This difference may have partly influenced the difference in CKD prevalence between our study and previous studies, with a slightly higher mean age of our study population also potentially playing a role.

Patients with CKD stage 3B or 4 (GFR 30–44 and 15–29 ml/min, respectively) had a decreased life expectancy of 17 and 25 years, respectively [20] in comparison with a decrease in life expectancy of 8 years in diabetes [21] and 3 years for hypertension [22]. CKD has recently been identified as a major risk factor for cardiovascular events [7, 8]. CKD and even proteinuria greatly increase cardiovascular risk and are considered the highest risk group for cardiovascular disease [2] and cardiovascular disease is the major cause of deaths in patients with CKD [23]. A study from Taiwan reported that cardiovascular disease accounted for 71% of deaths in patients with CKD, as compared to 22% in patients with normal kidney function [24]. We demonstrated that CKD in patients with hypertension associated with a substantially increased risk of cardiac and vascular complications including CAD, stroke, PAD, heart failure and atrial fibrillation. The adjusted Odds ratio was 1.4 in patients with GFR 30–59 ml/min and 1.7 for those with GFR below 30 ml/min, in comparison to those with GFR above 60 ml/min. This information is meaningful not only for specialists but also for primary care physicians [25]. We cannot make conclusion on the significance of proteinuria since the proteinuria data was relatively small. However, our data showed increased risk of cardiac and vascular complication of proteinuria in patients with CKD.

Many mechanisms facilitate and contribute to increased cardiovascular risk in CKD. Patients with CKD were commonly found to have concomitant cardiovascular risk factors, such as diabetes and hypertension. Patients with CKD had increased activation of renin-angiotensin and sympathetic response compared to those with normal renal function [8, 26]. Moreover, patients with CKD had higher levels of inflammatory biomarkers [27] and endothelial dysfunction [28]. In addition to increased risk of cardiovascular disease, patients with CKD, both with and without documented vascular disease, usually received treatment that was less than the treatment practice guidelines, as compared to patients without CKD [11].

Recent report shows a high prevalence of CKD in hypertensive patients and a strong association of CKD and cardiac and vascular complication [15]. This study also shows that more than half of patients with CKD are unaware of this condition. Therefore, these patients have limited opportunity to obtain advice on self-care to slow the progression of CKD and minimize the risk of complications. Registry data from several Asian countries show that a large proportion of CKD patients have a poor control of risk factors especially in those with diabetes [29].

Our study had some limitations. This was a cross-sectional study. We did not collect medication data such as anti-hypertensive medications, anti-thrombotic medications, and statins. The nature of our study design cannot prove cause and effect of identified associations. This study was designed for a cross-sectional survey and did not plan for a collection of follow-up data. Therefore we did not collect short or long-term outcome or changes in GFR or proteinuria. In fact, the main purpose of the main study was to determine the rate of blood pressure goal achievement and to feedback to the participating hospitals how good they are for blood pressure control. This study collected data from hospital based samples which may over-estimate the prevalence obtained from community based individuals. All patients were recruited from the ambulatory setting. Therefore the probability of recruiting acute kidney injury (AKI) cases should be very low since AKI cases should be admitted and managed in hospital setting. However, undetected AKI may be missed. The associations between cardiac and vascular complications with low cholesterol, low LDL-cholesterol, low triglyceride, and high HDL-cholesterol could be due to the effect of statin use, which would be more commonly found in patients with cardiac and vascular disease. This could make some results of our study difficult to interpret. Methods of creatinine measurement were not the same across all participating centers. Approximately half of the centers used enzymatic method for the measurement of serum creatinine and another half used modified Jaffe method. Enzymatic method was IDMS (isotope dilution-mass spectrometry)-traceable. Although the method that was used in many centers were not IDMS-traceable but all centers had their own laboratory standard quality control system that applied to local practice. Lastly, there were some cases with missing data, since it was not mandatory to have every variable entered into the case record form.

## Conclusion

In conclusion, prevalence of CKD in our hypertensive population was 37.5%. CKD is an independent risk factor that is associated with many cardiac and vascular complications. Improved awareness regarding detection and management of CKD is very important, especially for primary care physicians.

## Abbreviations

AF: Atrial fibrillation
CI: Confidence interval
CKD: Chronic kidney disease
CRF: Case record form
DBP: Diastolic blood pressure
DM: Diabetes mellitus
ECG: Electrocardiogram
EPI: Epidemiology collaboration formula
FPG: Fasting plasma glucose
GFR: Glomerular filtration rate
HDL: High density lipoprotein
LDL: Low density lipoprotein
MoPH: Ministry of public health
NHSO: National health security office
PAD: Peripheral arterial disease
SBP: Systolic blood pressure
SD: Standard deviation
TC: Total cholesterol
TG: Triglyceride
TIA: Transient ischemic attack
